# Role of In Vitro Fertilization (IVF) in Unexplained Infertility Management: A Systematic Review

**DOI:** 10.7759/cureus.72527

**Published:** 2024-10-28

**Authors:** Nadin A Mahabbat, Taghreed A Khan, Maad F Elyas, Ahmed A Mahabbat, Ayman M Oraif

**Affiliations:** 1 Department of Obstetrics and Gynecology, King Abdulaziz Medical City, Women's Health Hospital, Riyadh, SAU; 2 Department of Obstetrics and Gynecology, Heraa General Hospital, Mecca, SAU; 3 Department of Obstetrics and Gynecology, International Medical Center, Jeddah, SAU; 4 Medicine, Ibn Sina National College, Jeddah, SAU; 5 Department of Obstetrics and Gynecology, King Abdulaziz University Hospital, Jeddah, SAU

**Keywords:** embryo transfer, infertility management, in vitro fertilization, treatment, unexplained infertility

## Abstract

Unexplained infertility presents a significant reproductive challenge globally, affecting couples who are unable to conceive despite comprehensive fertility evaluations. This condition carries emotional, physical, and economic burdens, with a moderate to high prevalence, underscoring the need for effective interventions. In vitro fertilization (IVF) has emerged as a crucial treatment option for unexplained infertility, offering a unique approach to conception when traditional diagnostic tests fail to identify specific causes. However, the efficacy, safety, and comparative benefits of IVF in this context warrant comprehensive evaluation through systematic reviews. A systematic review was conducted to assess the effectiveness and outcomes of IVF in cases of unexplained infertility. Inclusion criteria encompassed peer-reviewed studies focusing on human subjects diagnosed with unexplained infertility and evaluating IVF outcomes.

Comprehensive searches were performed across electronic databases, and eligible studies were selected based on predetermined criteria. Data extraction was conducted independently by two researchers, and quality assessment was performed using standardized tools. The search yielded 10 studies comprising 73,884 patients, published between 2000 and 2021. Variability in methodological quality was observed across randomized controlled trials, while cohort studies demonstrated consistently high quality. Studies assessed outcomes like pregnancy success rate (46.2-47.9%), live birth rates (29.4-49%), time to pregnancy (9-14 months), cost-effectiveness, and adverse events such as ovarian hyperstimulation syndrome. Elective single embryo transfer (eSET) was found effective, and donor oocytes had higher live birth rates than autologous cycles. Multiple gestations and canceled IVF cycles were common. The systematic review underscores the complexity of IVF treatment for unexplained infertility, with variability in outcomes influenced by multiple factors. Strategies such as eSET and donor oocyte cycles show promise in optimizing treatment efficacy and safety. However, challenges such as heterogeneity, moderate to high risk of bias across studies, and cost considerations warrant careful interpretation and further research.

## Introduction and background

Unexplained infertility is a perplexing reproductive challenge affecting couples worldwide, characterized by the inability to conceive despite standard fertility evaluations yielding no discernible cause. It is typically diagnosed after comprehensive assessments, including hormonal testing, semen analysis, imaging studies (such as ultrasound or hysterosalpingography), and ovulation monitoring. When these evaluations do not identify specific abnormalities in reproductive function, the infertility is categorized as unexplained [1]. This enigmatic condition constitutes a significant global burden, impacting individuals emotionally, physically, and economically. The prevalence of unexplained infertility varies across regions and populations, but it is estimated to affect a substantial portion of couples seeking fertility assistance. According to global fertility studies, approximately 10-30% of couples experiencing infertility receive a diagnosis of unexplained infertility after undergoing standard fertility assessments [2]. The prevalence of unexplained infertility was estimated to be 8-37% [3]. The prevalence in the Middle Eastern region indicated that the overall infertility in the area was 38.5%, out of which the percentage of unexplained infertility was estimated to be 22.6% [4]. This prevalence underscores the widespread nature of the condition, highlighting the need for effective interventions to address its impact on individuals and societies.

The emotional toll of unexplained infertility is profound and extends beyond the realms of medical diagnostics. Couples grappling with unexplained infertility often face psychological distress, anxiety, and feelings of frustration due to the lack of a clear explanation for their fertility challenges. The uncertainty surrounding the cause can exacerbate the emotional strain, leading to strained relationships and diminished quality of life for those affected [5,6]. From an economic perspective, unexplained infertility contributes to the rising costs associated with infertility treatments globally. Fertility treatments often involve substantial financial investments. The need for multiple cycles of treatment, coupled with the absence of a clear diagnosis guiding the treatment plan, can lead to increased financial strain for individuals and couples seeking fertility assistance [7]. The economic burden extends beyond the direct costs of medical interventions to include indirect costs associated with time off work, travel for medical appointments, and the emotional toll of fertility-related expenses. Addressing the global burden of unexplained infertility requires a comprehensive and multidisciplinary approach [8].

In vitro fertilization (IVF) stands as a groundbreaking assisted reproductive technology that has revolutionized the landscape of fertility treatments. This complex procedure involves the fusion of an egg and sperm outside the body in a laboratory setting, resulting in the formation of embryos [9]. IVF plays a crucial role in addressing various infertility issues, and its significance is particularly pronounced in cases of unexplained infertility, where traditional diagnostic tests fail to identify the specific cause of a couple's inability to conceive. Unexplained infertility poses a perplexing challenge for both couples and fertility specialists, as routine evaluations often yield inconclusive results. In such cases, IVF becomes a valuable option, offering a unique approach to conception that bypasses potential obstacles in the reproductive system [10]. The process begins with controlled ovarian stimulation to induce the development of multiple eggs. These eggs are then carefully retrieved from the woman's ovaries through a minor surgical procedure. The next critical step in IVF involves the fertilization of the retrieved eggs with sperm in a controlled laboratory environment. This controlled environment allows fertility specialists to closely monitor the fertilization process, providing insights into any potential issues that may have hindered natural conception [3].

Embryo selection is another key feature of IVF that holds particular relevance in cases of unexplained infertility. Fertility specialists can evaluate the quality of embryos and select the most viable ones for transfer to the woman's uterus. This strategic approach enhances the chances of successful implantation and pregnancy [11]. Additionally, advancements in reproductive technologies have introduced preimplantation genetic testing (PGT), allowing for the screening of embryos for genetic abnormalities before implantation. This extra layer of scrutiny is particularly advantageous for couples with unexplained infertility concerns about the genetic health of embryos [12]. IVF also provides a comprehensive platform for monitoring the entire reproductive process. From the development of follicles and the retrieval of eggs to the cultivation of embryos in the laboratory, the procedure allows fertility specialists to closely track each stage. This meticulous monitoring enhances the chances of success, especially when dealing with unexplained infertility, where elusive factors may impact conception [13].

IVF is transforming lives globally, with improved clinical and laboratory approaches leading to better outcomes. The use of pre-treatment biomarkers to personalize exogenous gonadotrophin doses, gonadotrophin-releasing hormone (GnRH) antagonists to prevent premature luteinizing hormone (LH) surges, the transition from human chorionic gonadotrophin (hCG) to GnRH agonists to induce an endogenous LH surge, and cycle segmentation by temporally separating stimuli have all been implemented. However, failure to adequately execute these techniques can lead to ovarian hyperstimulation syndrome (OHSS) [14]. Although generally safe, pharmacological ovarian stimulation for IVF is associated with considerable repercussions, the most serious of which is OHSS. While the specific etiology of this condition is unknown, it is distinguished by cystic growth of the ovaries and a fluid shift from the intravascular to the interstitial space as a result of enhanced capillary permeability. The clinical appearance can range in severity from mild forms with stomach discomfort and nausea to severe, life-threatening complications as observed in moderate and severe OHSS. The incidence of moderate to severe OHSS is believed to be around 1-5% in all IVF cycles, with an associated death of nearly one in 50,000 persons. Mild cases are rather common, affecting up to 5% of women undergoing IVF, while severe cases occur in only 0.5% of IVF cycles. Ovarian stimulation is an important aspect of the IVF treatment process and OHSS is a serious iatrogenic effect of ovarian stimulation, as it can be fatal in severe cases. Polycystic ovaries, whether or not associated with polycystic ovarian syndrome, a high antral follicle count such as, at a young age, and a history of OHSS all increase the likelihood of developing OHSS [15].

This systematic review is motivated by the need to comprehensively assess and synthesize existing evidence regarding the efficacy and outcomes of IVF in this specific subgroup of infertility cases. Given the prevalence of unexplained infertility and the growing reliance on IVF as a treatment option, a systematic review aims to collate and critically appraise available studies, shedding light on the collective findings in terms of success rates, complications, and factors influencing outcomes. By employing rigorous methodology and inclusion criteria, such a review can provide a consolidated and evidence-based overview, offering insights into the effectiveness of IVF as an intervention for unexplained infertility. This systematic approach not only informs clinical practice but also guides future research directions, contributing to the optimization of fertility treatments and the holistic care of couples experiencing unexplained infertility.

## Review

Material and methods

This systematic review is reported according to the Preferred Reporting Items for Systematic Reviews and Meta Analyses (PRISMA) guidelines [16].

Search Strategy

In July 2024, we conducted a comprehensive search across electronic databases, including PubMed, Scopus, Web of Science, and ScienceDirect for publication. The search strategy employed appropriate combinations of keywords related to IVF, effectiveness, safety, efficacy, and unexplained infertility. The MeSH term and key terms, along with their combinations (utilizing Boolean operators AND and OR), were applied during the search. The keyword search strategy was: (("Assisted reproductive techniques" OR "Invitro fertilization" OR IVF AND safety OR efficacy OR role OR impact AND ("unexplained infertility"[MeSH Terms] OR "unexplained infertility" OR infertility. The aim was to assess the efficacy, safety, and comparative benefits of IVF compared to other treatment modalities available for couples diagnosed with unexplained infertility. The objectives of the review were to evaluate success rates in achieving pregnancy with IVF, compare its effectiveness against alternative assisted reproductive technologies (ART) or conservative treatments, assess safety concerns, identify prognostic factors for IVF success in unexplained infertility cases, and provide recommendations for clinical practice and future research directions. Through a comprehensive analysis of the existing literature, this review aims to provide insights into the optimal utilization of IVF in the management of unexplained infertility, informing both clinicians and patients in decision-making processes.

Definition of Outcomes and Inclusion Criteria

Inclusion criteria for this systematic review encompassed studies published in peer-reviewed journals focusing on human subjects diagnosed with unexplained infertility. Specifically, the review included investigations into the efficacy, safety, and comparative benefits of IVF as a treatment modality. Studies reporting outcomes such as IVF success rates, including pregnancy, live birth, or ongoing pregnancy rates, time of pregnancy, and cost-effectiveness, were considered. Additionally, studies comparing IVF with other ART or conservative treatments for unexplained infertility were included. Safety outcomes of IVF treatment, multiple pregnancies, or other adverse events were also considered.

Accordingly, the search strategy in PubMed, for example, was conducted as given below.

Keywords and MeSH terms: ("In vitro fertilization" OR "IVF") AND ("unexplained infertility" OR "infertility") AND ("efficacy" OR "outcomes" OR "treatment").

Filters applied: Human studies, publication years from 2000 to 2021, and peer-reviewed articles.

Search strategy: The search string was structured as follows: (("Assisted reproductive techniques"[MeSH Terms] OR "In vitro fertilization"[MeSH Terms]) AND ("unexplained infertility"[MeSH Terms] OR "unexplained infertility")) AND (safety OR efficacy OR role OR impact).

Exclusion Criteria

To maintain precision and reliability, the exclusion criteria involved the exclusion of animal studies, in vitro investigations, laboratory studies, and research with redundant findings. Additionally, abstract-only presentations, reviews, books, posters, theses, editorials, notes, letters, case reports, case series, and conference papers were excluded. Studies were selected based on the inclusion and exclusion criteria by two independent authors. Any disagreement was settled by consensus among all authors.

Screening and Extraction

Articles with irrelevant titles were excluded from consideration. In the subsequent phase, both the full text and abstracts of the papers were meticulously reviewed to determine their compliance with the inclusion criteria. To streamline the process, titles and abstracts were organized, assessed, and scrutinized for any duplicate entries using reference management software (Endnote X8; Clarivate, London, United Kingdom). To ensure the highest quality of selection, a dual screening approach was adopted, involving one screening for the evaluation of titles and abstracts, and another for the comprehensive examination of the entire texts. Once all relevant articles were identified, a structured extraction sheet was created to capture pertinent information aligned with our specific objectives.

Two separate researchers conducted the data extraction process independently. The gathered information included various study attributes like the author's name, publication year, country of origin, study design, sample size, duration of follow-up, and sources of funding. Additionally, details regarding participants, such as age, gender, and nationality, were collected. Specifics about the interventions employed in the studies were documented, alongside outcome measures like the success rates of IVF, encompassing parameters such as pregnancy rates, live birth rates, and ongoing pregnancy rates, along with the timing of pregnancies. Furthermore, any information pertaining to cost-effectiveness was also included in the extraction process.

Quality Assessment

We employed the Cochrane Risk of Bias assessment tool to evaluate the methodological quality and risk of bias in randomized controlled trials (RCTs) included in this review [17]. Additionally, for cohort studies, we utilized the Newcastle-Ottawa Scale (NOS) to assess the quality and potential biases in these observational studies [18]. By employing these standardized tools, we aimed to rigorously evaluate the strength of evidence presented in the included studies and ensure the reliability of our findings. This approach allows for a comprehensive assessment of both randomized and observational studies, enhancing the robustness and validity of our systematic review.

Results

Search Results

**Figure 1:**
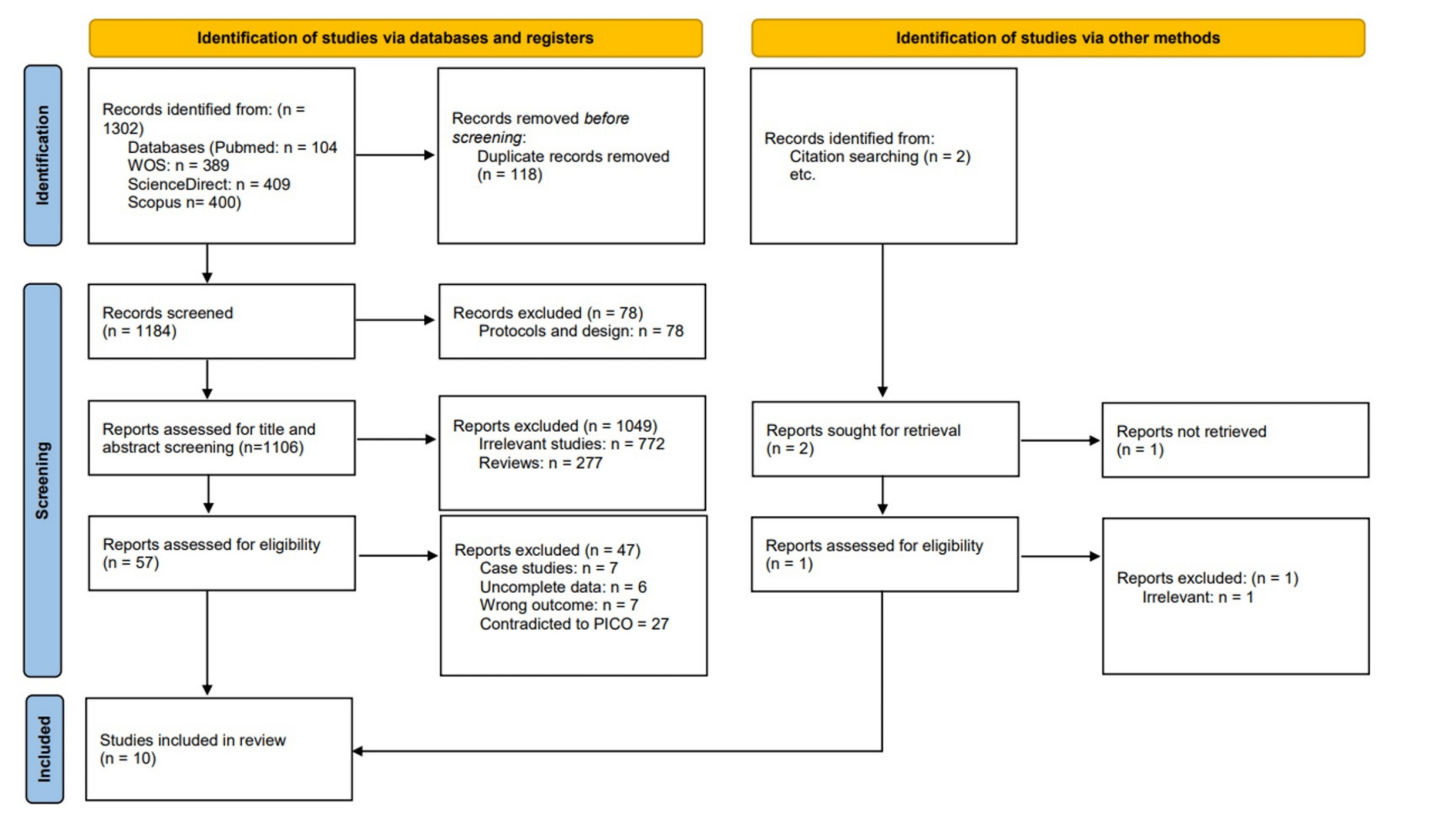
Preferred Reporting Items for Systematic Reviews and Meta-Analyses (PRISMA) flow diagram

We executed the search methodologies outlined previously, resulting in the identification of a total of 1302 citations, subsequently reduced to 1184 following the removal of duplicates. Upon screening titles and abstracts, only 57 citations met the eligibility criteria for further consideration. Through full-text screening, this number was further refined to 10 articles [19-28] aligning with our inclusion and exclusion criteria. Figure 1 provides an in-depth depiction of the search strategy and screening process.

Results of Quality Assessment

Cochrane risk of bias assessment for RCTs results revealed variability in methodological quality across studies. While some, like Nandi et al. [19] and Goldman et al. [20], showed low risk in most domains, others like Van Rumste et al. [21] and Foong et al. [22] exhibited unclear or high risk, suggesting potential biases. Hughes et al. [23] and Goverde et al. [24] also displayed mixed results. Methodological limitations and reporting biases emphasize the need for a cautious interpretation of findings and highlight the importance of further research with improved rigor.

On the other hand, the NOS assessment for cohort studies resulted in consistently high scores across the studies evaluated. Wessel et al. [25], Koot et al. [26], van Eekelen et al. [27], and Yeh et al. [28] all demonstrated good quality in terms of selection, comparability, and outcome assessment. This suggests robust methodology and reporting practices in these cohort studies, enhancing the reliability of their findings. The detailed findings of the quality assessment are presented in Tables 1, 2.

**Table 1:** Cochrane risk of bias assessment for randomized controlled trials

Study	Random sequence generation	Allocation concealment	Blinding of participants and personnel	Blinding of outcome assessment	Incomplete outcome data	Selective reporting	Other bias	Overall
Nandi A et al. (2017) [19]	Low	Low	High	High	Low	Low	Low	Moderate
Goldman MB et al. (2014) [20]	Low	Low	High	Low	Low	Low	Low	Moderate
Van Rumste MM et al. (2014) [21]	Unclear	Unclear	High	High	Low	Low	Low	High
Foong SC et al. (2006) [22]	Unclear	Unclear	High	High	Low	Unclear	High	High
Hughes EG et al. (2004) [23]	Unclear	Low	High	High	Low	High	Low	Moderate
Goverde AJ et al. (2000) [24]	Unclear	Unclear	Unclear	High	Low	Low	High	High

**Table 2:** Newcastle-Ottawa Scale of each included cohort study

Study	Selection	Comparability	Outcome	Total score	Quality
Wessel JA et al. (2021) [25]	3	2	2	7	Good
Koot YE et al. (2019) [26]	4	2	2	8	Good
van Eekelen R et al. (2019) [27]	4	1	4	9	Good
Yeh JS et al. (2014) [28]	4	2	1	7	Good

Characteristics of the Included Studies

We incorporated a total of 10 studies encompassing 73,884 patients, published between 2000 and 2021. All participants in these studies were aged 18 to 40 years. Notably, all the studies were RCTs or cohort studies. Geographically, three studies originated from the United Kingdom and the other three from the Netherlands, followed by two studies each from the United States and Canada. A comprehensive summary of the baseline characteristics of these studies can be found in Table 3. Discrepancies in sample sizes across the included papers likely stem from differences in study objectives and inclusion criteria.

**Table 3:** Summary of baseline characteristics of studies NR: Not reported; RCT: Randomized controlled trial; IVF: In vitro fertilization; IVF–e SET:  elective single-embryo transfer; NIEKS: National Institute of Eunice Kennedy Shriver, NICH: National Institute of Child Health, HD: Human Development

Author	Year	Registration	Country	Study design	Study period	Total participants	Mean age of females (IVF)	Funding
Wessel JA et al. [25]	2021	NL915 (NTR939); NL3895 (NTR4057)	Netherlands	Prospective cohort	NR	472, IVF=138	39.3 ±1.4	The Dutch Organization for Health Research and Development (ZonMW)
Koot YE et al. [26]	2019	NR	UK	Retrospective cohort	2008 and 2012	118	39 years	The University Medical Center Utrecht, in Utrecht, and the Academic Medical Centre, in Amsterdam
van Eekelen R et al. [27]	2019	NR	UK	Retrospective cohort	1999- 2011	Cohort 1=40921, cohort 2=4875, and cohort 3=975	Cohort 1=35.1 years, cohort 2=32.5 years, cohort 3= 33.2 years	Tenovus Scotland travel (grant G17.04), supported by the Amsterdam. Reproduction & Development Research Group (grant V.000296 to RvE)
Nandi A et al. [19]	2017	ISRCTN43430382	UK	RCT	2013 - 2015	207 IVF = 106	Median (IQR): 32.5 (30–35)	NR
Goldman MB et al. [20]	2014	NCT00246506	USA	RCT	2004-2009 and 2008-2009	154, IVF=51	39.9±1.4	Supported by NIEKS, NICH, and HD (grant R01-HD44547)
Van Rumste MM et al. [21]	2014	ISRCTN86744378	Multicenter, Netherlands	RCT	2006 - 2009	116, IVF–e SET=58	34 years	An unconditional grant from Organon
Yeh JS et al. [28]	2014	NR	US	Retrospective cohort	2008- 2010	26,457 cycles, donor oocytes / autologous cycles: 11,420 / 15,037	25.4±2.6/ 28.0±2.0	NR
Foong SC et al. [22]	2006	NR	Canada	RCT	1997 and 2001	60, IVF = 30	18–40 years	NR
Hughes EG et al. [23]	2004	NR	Canada	Clinical trial	2000 and 2002	139, IVF=68	32.9±3.2 years	NR
Goverde AJ et al. [24]	2000	NR	Netherlands	Clinical trial	NR	258, IVF=87	NR	NR

Study Outcome Measures

Several studies assessed various aspects of ART such as pregnancy success rate, live birth rate, time to pregnancy, cost-effectiveness, and adverse events. Wessel et al. [25] reported a 29.4% live birth rate with no details on pregnancy success rate or time to pregnancy. Koot et al. [26] demonstrated a live birth rate of 49%, with a median time to pregnancy of nine months and a mean of 14 months. Van Eekelen et al. [27] found a pregnancy success rate of 47.9%, with a one-year chance of conception difference of 21.8%, along with a notable rate of multiple gestations and canceled IVF cycles. Nandi et al. [19] and Goldman et al. [20] exhibited live birth rates of 46.2% and 49.0%, respectively, with varying costs per live birth and occurrences of adverse events such as ovarian hyper-stimulation syndrome. Van Rumste et al. [21] presented ongoing pregnancy rates and costs associated with IVF, highlighting the effectiveness of elective single embryo transfer (eSET). Yeh et al. [28] compared outcomes between donor oocytes and autologous cycles, revealing significant differences in live birth rates. Foong et al. [22] and Hughes et al. [23] reported pregnancy success rates and live birth rates, with Hughes et al. noting the impact of embryo transfer on multiple pregnancies. Lastly, Goverde et al. [24] reported a lower pregnancy success rate with no details on adverse events or cost-effectiveness. These findings collectively underscore the multifaceted considerations involved in ART, including success rates, safety, and cost implications (Table 4).

**Table 4:** Summary of outcomes of included studies RR: Relative risk ratio; CI: Confidence interval; OR: Odds ratio; NR: Not reported; IVF: In vitro fertilization

Author	Pregnancy success rate	Live birth rate	Time to pregnancy	Cost-effectiveness	Adverse events
Wessel JA et al. [25]	NR	29.24%	NR	NR	NR
Koot YE et al. [26]	NR	49% (95% CI: 39–59%)	median 9 months and mean 14 months (95% CI: 10–18)	NR	NR
van Eekelen R et al. [27]	47.9%, difference of average adjusted 1-year chances of conception. : 21.8% (95%CI: 18.3–25.3)	NR	1 year	NR	Multiple gestations: In the UK IVF cohort, 30% resulted in multiple gestations.
Nandi A et al. [19]	46.2%, multiple: 8.3%	31.1%	NR	All cycles: £316,800, per live birth: £10,560.00	In the IVF group, there were 3 cases of ovarian hyperstimulation syndrome.
Goldman MB et al. [20]	49.0%	84.2%	8.7 ± 0.5 months,	NR	NR
Van Rumste MM et al. [21]	Ongoing pregnancy=24%, multiple=14%	22%	NR	€2781; additional ongoing pregnancy: €2456	NR
Yeh JS et al. [28]	Donor oocytes vs. autologous cycles 68% / 59%, OR (CI): 1.57 (1.27–1.95), p<0.001	60% / 53%, OR (CI): 1.47 (1.20–1.81), p<0.001	NR	NR	NR
Foong SC et al. [22]	50%	46.7%	NR	NR	NR
Hughes EG et al. [23]	31%	29%	NR	NR	NR
Goverde AJ et al. [24]	12.2%	NR	NR	Dutch guilders (US$14679)	NR

Discussion

This study aimed to evaluate the effectiveness and outcomes of IVF, specifically in cases of unexplained infertility, by synthesizing existing evidence. Results from our review indicate that success rates of ART procedures, safety considerations, and cost implications collectively influence treatment outcomes. The proportion of live births across the studies exhibited notable variability, alongside variations in the median time required for pregnancy and the incidence of adverse events. Furthermore, the efficacy of strategies like eSET and the utilization of donor oocytes versus autologous cycles were evident.

The success of ART in treating unexplained infertility is a crucial area of study, with numerous investigations contributing valuable insights. Typically gauged through live birth rates, ART success rates in this context exhibit notable variability, reflecting the complexity of unexplained infertility and variations in patient demographics, treatment protocols, and ART procedures across studies [29]. Variations in live birth rates among studies can be a result of patient characteristics like age, ovarian reserve, and infertility duration, which significantly influence the treatment outcomes [3,30,31]. Advanced maternal age and diminished ovarian reserve correlate with reduced ART success rates, including lower live birth rates. Moreover, treatment protocol differences, encompassing ovarian stimulation regimens, embryo transfer techniques, and laboratory protocols, also impact outcomes [32]. Some protocols prove more effective in specific patient subsets, contributing to outcome variability. Additionally, adjunctive procedures, such as preimplantation genetic testing, influence live birth rates but vary in utilization across studies [33].

eSET emerges as a beneficial strategy in managing unexplained infertility, supported by extensive literature. It maximizes pregnancy chances while minimizing multiple gestation risks [34]. Transferring a single embryo reduces the multiple pregnancies likelihood, associated with heightened maternal and neonatal complications [35]. eSET also yields comparable or superior live birth rates to multiple embryo transfers in specific patient subsets, including those with unexplained infertility. Moreover, eSET decreases preterm birth, low birth weight, and neonatal intensive care unit admissions, emphasizing its safety and efficacy [34,36]. Aligned with personalized medicine principles, eSET tailors treatment to individual patient preferences and characteristics [37]. Another concern is that comparative literature analysis highlights donor oocyte cycles' superior efficacy over autologous cycles in unexplained infertility treatment. Donor oocytes provide youthful egg quality, enhancing embryo development and implantation rates. By bypassing autologous cycle egg quality or quantity issues, donor oocyte cycles increase conception and live birth likelihood [38,39]. Nonetheless, donor gamete use entails emotional, psychological, and ethical considerations. Autologous cycles offer genetic-related benefits but may be less cost-effective and accessible. Considering patient preferences and ethical aspects helps choose the most suitable treatment approach [38].

Similar to our findings, the results of a meta-analysis from recent times defined that there is no robust evidence that in couples with unexplained infertility, IVF yields pregnancy and live birth faster than intrauterine insemination (IUI)-ovarian stimulation. Both IVF and IUI-ovarian stimulation are effective and safe treatments for managing unexplained infertility. The associated costs of interventions and couples' preferences must be considered in clinical decision-making [40]. Another review of present times also concluded that scientific evidence suggests that IVF should be used as first-line management, but the scarcity of high-quality RCTs, combined with the heterogeneity of the identified studies and a lack of research among women over 40 years, highlights the need for additional large, randomized trials. The decision to provide IUI with ovarian stimulation or IVF should be based on patient prognosis. Hence, the authors recommended IUI ovarian stimulation as the first-line treatment for unexplained infertility in women under 38 years old with a good prognosis, while IVF should be administered initially to those over 38 years old. Patients should be appropriately counseled to allow for informed decision-making [41].

Adding more evidence in this context, findings from another systematic review suggested that IVF may be associated with higher live birth rates than expectant management; however, there is inadequate evidence to make definite recommendations. IVF may also result in greater live birth rates than unstimulated IUI. In women who have had clomiphene and IUI, IVF appears to be related to greater birth rates than IUI with gonadotropins. In treatment-naive women, there is no significant difference in live birth rates between IVF, IUI + gonadotropins, or IVF + clomiphene [42]. However, Sunkara et al. demonstrated in their review findings that there may be minimal or no difference in live birth rates between IVF and IUI + gonadotropins, or IVF and IUI + clomiphene citrate. Assuming a 42% live birth rate with IUI + gonadotropins and a 26% live birth rate (1 IVF to 1 IUI cycle), the live birth rate with IVF would be 39% to 54% and 24% to 51%, respectively. Assuming a 15% live birth rate with IUI and clomiphene citrate, the live birth rate with IVF would range from 15% to 54% [43].

The most effective method of managing unexplained infertility in couples remains unclear, and the clinical question is how to optimize the patient's outcomes. Hence, it is recommended to consider resources, patient age, and the duration of infertility when developing a treatment strategy based on the best available evidence [3]. Safety considerations are further critical in ART for unexplained infertility, OHSS, and multiple gestations primary concerns. OHSS risks, mitigated through antagonist protocols and modified gonadotropin dosing, underscore the need for careful ovarian stimulation management [44]. Multiple gestation hazards, including preterm birth and neonatal complications, necessitate strategies to minimize such risks. Long-term health outcome monitoring ensures ART-conceived children's well-being [45,46].

OHSS was first recognized over six decades ago and continues to remain a serious complication associated with ovarian stimulation with gonadotropins, particularly in IVF cycles, additionally leading to financial strain [47]. Mild OHSS is classified into grades, starting with Grade 1, which includes abdominal distension and discomfort. Grade 2 encompasses all Grade 1 symptoms, along with nausea, vomiting, diarrhea, and ovarian enlargement ranging from 5 to 12 cm. Moderate OHSS, or Grade 3, includes the symptoms of mild OHSS with the addition of ultrasonographic evidence of ascites. Severe OHSS is also classified into Grade 4, which features moderate symptoms along with clinical signs of ascites and/or hydrothorax and breathing difficulties, and Grade 5, which includes all previous symptoms plus significant changes in blood volume, increased blood viscosity due to hemoconcentration, coagulation issues, and reduced renal perfusion and function [48].

OHSS mainly develops in individuals who have an intense reaction to exogenous gonadotropins when they receive hCG to complete oocyte maturation, leading to the development of multiple corpora lutea. The extended half-life of hCG compared to endogenous LH results in continuous luteotropic activity, generating vasodilation, increased capillary permeability, and fluid shift from intravascular to extravascular regions third space, resulting in hypovolemic hyponatremia. Clinically, OHSS is defined by ovarian cystic growth, abdominal distention and pain, and fluid transfer from the intravascular to the third space, which can result in ascites, pericardial and pleural effusions, and generalized edema. Adult respiratory distress syndrome, thromboembolism, and acute renal failure are all potential life-threatening complications of OHSS [47]. While gonadotrophin stimulation leads to ovarian enlargement, the cause of increased vascular permeability associated with OHSS is unknown. The concept that high levels of estrogen cause greater vascular permeability has been disagreed with. Hyperpermeability is likely caused by vascular endothelial growth factor, which has a strong correlation with hCG levels. Severity ranges from asymptomatic to ovarian enlargement with cysts, nausea, shortness of breath, and pleurisy. Symptoms of severe OHSS include bloating due to ovarian enlargement and the development of ascitic fluid in the peritoneum [49].

OHSS is a serious iatrogenic complication associated with ART, making it crucial to identify its risk factors and implement timely preventive strategies [50]. Preventing OHSS is preferred over treating it and involves assessing various risk factors, including young age, low body mass index, polycystic ovarian syndrome, high gonadotropin doses, and a history of previous OHSS. Additional risk factors include a high number of growing follicles on the day of trigger, a large number of oocytes retrieved, and elevated serum estradiol levels at the time of triggering. For patients at risk of developing OHSS, controlled ovarian stimulation protocols using GnRH antagonists followed by GnRH agonist triggers and freeze-all strategies are recommended to reduce the likelihood of severe cases [51]. Modifiable predictors of OHSS include the number of oocytes extracted, pregnancy following fresh embryo transfer, and the type of drug utilized for pituitary suppression during controlled ovarian hyperstimulation. Patients with OHSS have a greater risk of preterm delivery and poor birth weight. Therefore, clinicians should minimize the risk of OHSS wherever possible [52].

Cost implications also heavily influence ART management for unexplained infertility. The analysis of included studies reveals a substantial range in costs associated with IVF cycles. For instance, Nandi et al. reported that average cost per live birth was about $10,560 and the total cost for all IVF cycles amounted to approximately £316,800, highlighting significant financial implications for patients. Similarly, Goverde et al. noted costs of approximately $14,679 for IVF cycles, indicating a varying economic burden across different healthcare systems. Treatment cycle expenses, compounded by multiple cycle needs, strain finances. Age, ovarian reserve, and prior treatment outcomes affect the likelihood of subsequent cycle success, complicating cost considerations [7,53]. Adjunctive procedures' cost-effectiveness warrants careful evaluation against potential benefits. Indirect costs, such as lost wages and emotional toll, further burden couples. Hence, financial planning and consideration of both direct and indirect costs are essential for informed decision-making regarding treatment options [54].

Strengths and Limitations 

The present systematic review assesses various outcomes, such as pregnancy success rates, live birth rates, time to pregnancy, cost-effectiveness, and safety considerations. Despite some methodological variability among studies, rigorous quality assessments reveal consistent high-quality evidence, particularly in cohort studies. The findings highlight the complexity of IVF treatment for unexplained infertility, showcasing variability influenced by patient demographics, treatment protocols, and adjunctive procedures. Strategies like eSET and donor oocyte cycles demonstrate promise in enhancing treatment efficacy and safety. Overall, the systematic review provides valuable insights into optimizing IVF treatment for couples with unexplained infertility, contributing to improved patient care and outcomes.

Despite the valuable insights provided, the systematic review assessing the role of IVF in treating unexplained infertility is not without limitations. Firstly, the inclusion criteria may introduce selection bias, as studies meeting specific eligibility criteria may omit relevant research, potentially affecting the comprehensiveness of the review. Additionally, heterogeneity across included studies, such as variations in patient demographics, treatment protocols, and outcome measures, complicates data synthesis and interpretation. This heterogeneity may limit the ability to draw definitive conclusions or generalize findings across diverse patient populations. A key limitation is the inclusion of studies with a moderate to high risk of bias. This may weaken the reliability of the findings and the overall strength of the conclusions. However, these were the only studies with full-length aligning with the inclusion and exclusion criteria. While including these studies was necessary to provide a comprehensive overview of available evidence on IVF, it is important to acknowledge that their risk of bias necessitates a cautious interpretation of the results. 

Furthermore, the quality of evidence across studies may vary, with discrepancies in study design, sample size, and methodology affecting the reliability and robustness of findings. Variability in outcome reporting and insufficient data on important parameters, such as pregnancy outcomes and adverse events, may also limit the review's ability to provide comprehensive insights into IVF's role in treating unexplained infertility. Moreover, the potential for publication bias, where studies with positive results are more likely to be published than those with negative findings, may skew the overall conclusions of the review. Finally, the dynamic nature of IVF technology and evolving treatment protocols may render some included studies outdated or less relevant to current clinical practice, necessitating careful consideration of the review's findings within the context of contemporary ART approaches for unexplained infertility.

Future Research Implications and Recommendations

Future research in the realm of IVF for treating unexplained infertility should prioritize several key areas to advance knowledge and improve clinical practice. Prospective studies with robust methodologies, expanded geographical scope, larger sample sizes, and low risk of bias are needed to further elucidate the effectiveness of IVF in this patient population. These studies should aim to standardize treatment protocols and outcome measures to facilitate comparison and meta-analysis across studies. Additionally, there is a need for research focusing on identifying predictive factors for IVF success in patients with unexplained infertility, such as biomarkers of ovarian reserve or genetic markers associated with embryo implantation. Furthermore, comparative effectiveness studies comparing different IVF treatment strategies, such as the use of elective eSET versus multiple embryo transfer, would provide valuable insights for optimizing treatment outcomes while minimizing risks.

Long-term follow-up studies assessing the health outcomes of children conceived through IVF in cases of unexplained infertility are also warranted to address concerns regarding the safety and well-being of ART-conceived offspring. Moreover, research investigating the psychosocial and economic impact of IVF treatment on couples with unexplained infertility is essential to inform supportive interventions and improve patient-centered care. Finally, interdisciplinary collaborations between clinicians, researchers, and patients are crucial for translating research findings into clinical practice and addressing the multifaceted challenges associated with IVF treatment for unexplained infertility. By prioritizing these research directions and recommendations, the effectiveness, safety, and accessibility of IVF for couples struggling with unexplained infertility can be significantly enhanced.

## Conclusions

This systematic review highlights IVF as a crucial treatment option for unexplained infertility and its complexity in treatment, with variability in outcomes influenced by multiple factors. The majority of the studies in this review demonstrated modest rates of successful pregnancy and live birth following IVF; however, challenges such as heterogeneity, moderate to high risk of bias across studies, and cost considerations warrant careful interpretation of these results. Despite variations in success rates and treatment outcomes across studies, IVF remains a feasible option, presenting a hopeful prospect. Optimizing IVF treatment for couples with unexplained infertility contributes to improved patient care and outcomes. Strategies such as eSET and donor oocyte cycles show promise in optimizing treatment efficacy and safety. Further research is essential to establish evidence-based guidelines for the utilization of IVF in the management of unexplained infertility, specifically to enhance patient outcomes.
